# Neuronal migration genes and a familial translocation t (3;17): candidate genes implicated in the phenotype

**DOI:** 10.1186/s12881-020-0966-9

**Published:** 2020-02-06

**Authors:** Meriam Hadj Amor, Sarra Dimassi, Amel Taj, Wafa Slimani, Hanene Hannachi, Adnene Mlika, Khaled Ben Helel, Ali Saad, Soumaya Mougou-Zerelli

**Affiliations:** 1 Department of Human Cytogenetics, Molecular Genetics and Reproductive Biology Farhat Hached University Teaching Hospital, Ibn El Jazzar street, 4000 Sousse, Tunisia; 2 High Institute of Biotechnology, Monastir University, 5000 Monastir, Tunisia; 3 Common Service Units for Research in Genetics, Faculty of Medicine of Sousse, University of Sousse, Ibn El Jazzar street, 4000 Sousse, Tunisia; 4 Pediatric department, Farhat Hached University Teaching Hospital, Ibn El Jazzar street, 4000 Sousse, Tunisia; 5 Pediatric department, Ibn Jazzar University Teaching Hospital, Ibn El Jazzar Street, 3100 Kairouan, Tunisia

**Keywords:** *CHL1*, Miller-Dieker syndrome critical region, *PAFAH1B1*, Partial monosomy 3p26.2, Partial trisomy 17p13.3

## Abstract

**Background:**

While Miller-Dieker syndrome critical region deletions are well known delineated anomalies, submicroscopic duplications in this region have recently emerged as a new distinctive syndrome. So far, only few cases have been described overlapping 17p13.3 duplications.

**Methods:**

In this study, we report on clinical and cytogenetic characterization of two new cases involving 17p13.3 and 3p26 chromosomal regions in two sisters with familial history of lissencephaly. Fluorescent In Situ Hybridization and array Comparative Genomic Hybridization were performed.

**Results:**

A deletion including the critical region of the Miller-Dieker syndrome of at least 2,9 Mb and a duplication of at least 3,6 Mb on the short arm of chromosome 3 were highlighted in one case. The opposite rearrangements, 17p13.3 duplication and 3p deletion, were observed in the second case. This double chromosomal aberration is the result of an adjacent 1:1 meiotic segregation of a maternal reciprocal translocation t(3,17)(p26.2;p13.3).

**Conclusions:**

17p13.3 and 3p26 deletions have a clear range of phenotypic features while duplications still have an uncertain clinical significance. However, we could suggest that regardless of the type of the rearrangement, the gene dosage and interactions of *CNTN4, CNTN6* and *CHL1* in the 3p26 and *PAFAH1B1, YWHAE* in 17p13.3 could result in different clinical spectrums.

## Background

The diagnosis of human chromosome abnormalities including gain or loss of genomic copy numbers has extremely benefited from the development of advanced molecular cytogenetic methods such as array-CGH. This allows high-resolution pangenomic analysis, in particular in detecting genetic imbalances, defining their size, delimiting translocation breakpoints and analyzing the involved segments [1]. Array-CGH has identified novel co-locating micro-deletions and micro-duplication in the same locus. This has allowed the description of new genomic disorders leading to distinct clinical phenotypes. Recently, the duplication of the entire Miller-Dieker syndrome critical region (MDS) involving *PAFAH1B1* and *YWHAE* genes as well as new co-locating micro-duplications in chromosome 17p13.3 have been defined within duplication syndromes in the MDS locus [2, 3]. Likewise, deletions and duplications of 3p26 region have been described as new emerging syndromes [4–6].

In this study, we report a familial translocation (3;17) leading to two different cytogenetic rearrangements resulting in a duplication/deletion of the 17p13.3 critical region for MDS including *PAFAH1B1* and *YWHAE* genes and 3p26 region including *CNTN4, CNTN6, CRBN* and a part of *CHL1*. The duplication and deletion of the same chromosomal region resulted as expected in distinct phenotypic features in the offspring.

## Methods

### Clinical report

#### Patient1 (the proband)

**Fig. 1:**
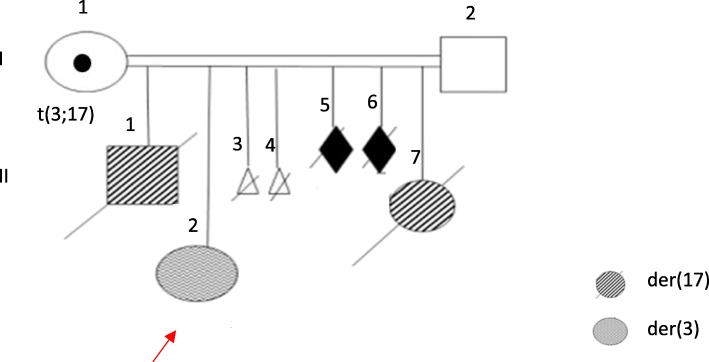
Pedigree of the family

**Fig. 2:**
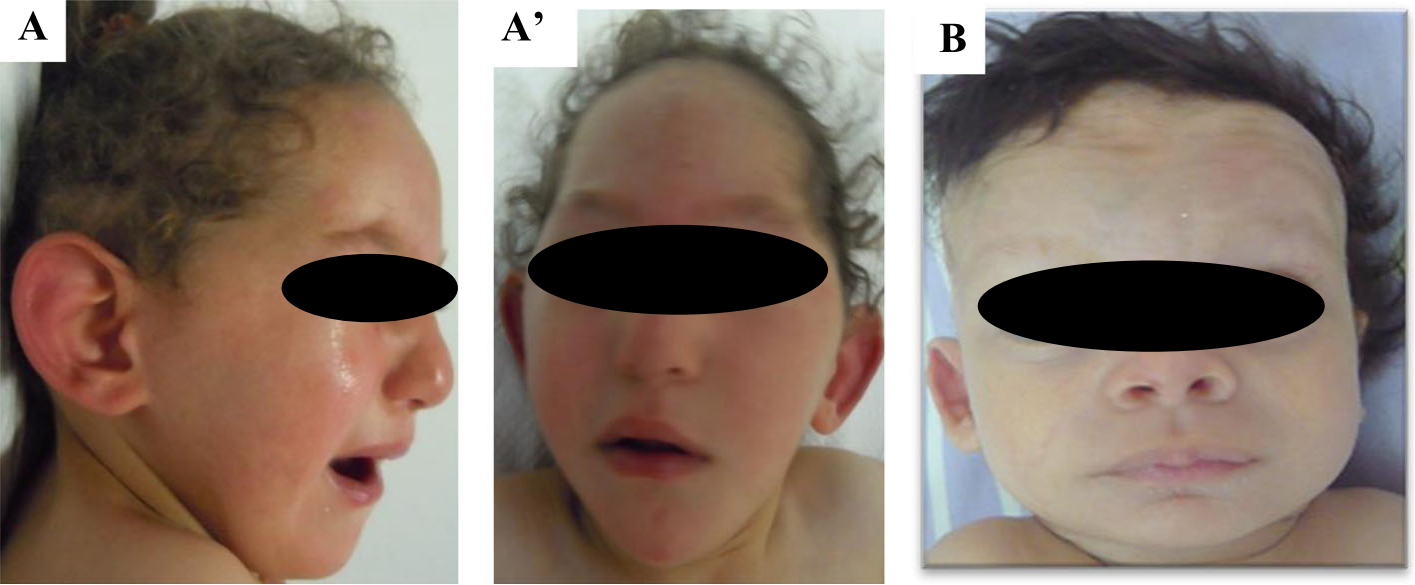
Photographs of the patients

A 2-year-old girl referred for the cytogenetic exploration with a family history of lissencephaly (Fig. 1.II-2), is the second child of a healthy consanguineous Tunisian couple. The patient’s weight at birth was 3500 g (+ 0,6SD). She measured 52 cm (+ 1,05SD) and had a head circumference of 35 cm (+ 0,4SD). At 2 years of age, her height and head circumference were 88 cm (+ 0,9SD) and 45 cm (− 2,5SD), respectively. At physical examination, she had psychomotor development delay and an abnormal behavior including aggressiveness, anger and agitation. Furthermore, she had craniofacial dysmorphic features (Fig. 2a, A’) including a long face, a high forehead, down-slanting palpebral fissures, epicanthus, a wide nose, a long philtrum, a thin upper lip, large and high implanted ears and a pointed chin with micrognathia. In addition, she showed arachnodactyly. Her cerebral magnetic resonance imaging (MRI) was performed at two years and five months of age, and corpus callosum hypoplasia was detected.

#### Patient 2

The patient (Fig. 1.II-7) presented at 4 months for exploration because of growth retardation, axial hypotonia, seizure and dysmorphic features (Fig. 2b) including a high forehead, a wide nose, low implanted ears and lissencephaly at MRI. She died 10 months later. Her brother (Fig. 1.II-1) suffering from type 1 lissencephaly, also died at an early age.

The proband (II-2) (gray) and her sister (II-7) (striped) carried a der (3) and der (17) respectively. The white triangle and the black diamond represent terminated pregnancies and affected stillborn, respectively.

### Karyotype

Metaphase chromosome preparations were obtained by phytohemagglutinin (PHA) stimulated lymphocyte culture according to standard procedures. Chromosome analysis was carried out applying R-banding at a 500-band level according to ISCN 2016 [7] in the patient, parents and sister.

### Fluorescent in situ hybridization (FISH)

FISH was performed on blood lymphocytes blocked on metaphases of the patient (II-2), those of her sister (II-7) and those of her mother, according to the standard protocol. One probe screening the chromosome 17 short arm was used: commercial probes; Miller-Dieker/Lissencephaly region probe set: LISI (Red) and RARA (Green) (Vysis) (Abbott Laboratories, IL, USA).

The hybridized chromosomal spreads were analyzed using a fluorescent microscope equipped with appropriate filters and Cytovision FISH system image capture software (Zeiss Axioskop 2 plus). Slides were scored on the basis of the number of probe signals for each metaphase. For each target area ten hybridized metaphases were analyzed.

### Array CGH

Oligonucleotide array CGH was performed using the Agilent Human Genome CGH Microarray Kit 44 K®. This microarray consisted of more than 44,000 oligonucleotide probes that spanned both coding and non-coding regions. The coverage of the human genome was made with an average spatial resolution of 75,000 pair bases.

The patient’s DNA as well as a reference DNA was fragmented by heat at 95 °C for 20 min. Each fragmented DNA product was labeled by random priming using either ULS5 or ULS3. After column-purification, probes were denatured and pre-annealed with 5 μg of human Cot-1 DNA, 10 μl of CGH Blocking agent and 55 μl of hybridization buffer. Hybridization was performed at 65 °C during 24 h. The microarray was washed, scanned and analyzed with Agilent Feature Extraction® 9.1 software. Results were interpreted using DNA analytics® 4.5 software. Only imbalances involving three or more adjacent probes were held. The identification of probes with a significant gain or loss was based on the log^2^ ratio plot deviation from 0 with cutoff values of 0.5 to 1, and − 0.5 to − 1, respectively.

## Results

The conventional cytogenetic analysis did not reveal any chromosomal anomalies in the two sisters (II-2/II-7) nor in parents’ karyotypes.

**Fig. 3:**
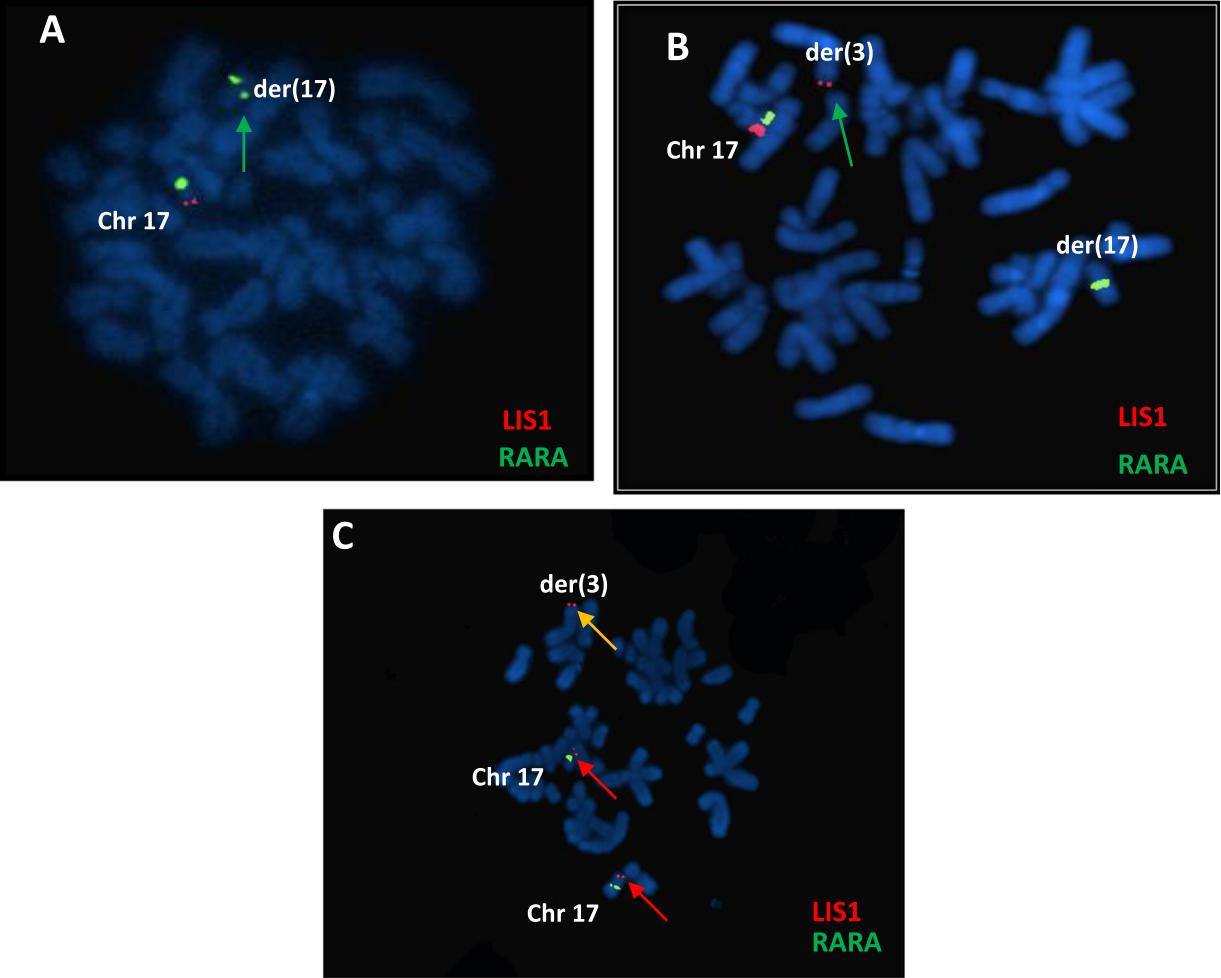
FISH analyses. **a** FISH results from patient II-7 using commercial Miller Dieker/Lissencephaly region probe set: (Lsi LIS1: Red and Lsi RARA: Green) showing the absence of the red fluorescence signal on the arrowed der(17), suggesting that the *LIS1* gene is deleted. **b** FISH results from mother using the same commercial probe, demonstrating the translocation of terminal material from 17p to chromosome 3p (green arrow). **c** FISH results from patient II-2 using the commercial Miller Dieker/Lissencephaly region probe set showing the presence of three red fluorescence signal on the arrowed der(3) and the two arrowed chr 17, confirming that the *LIS1* gene is duplicated

**Fig. 4:**
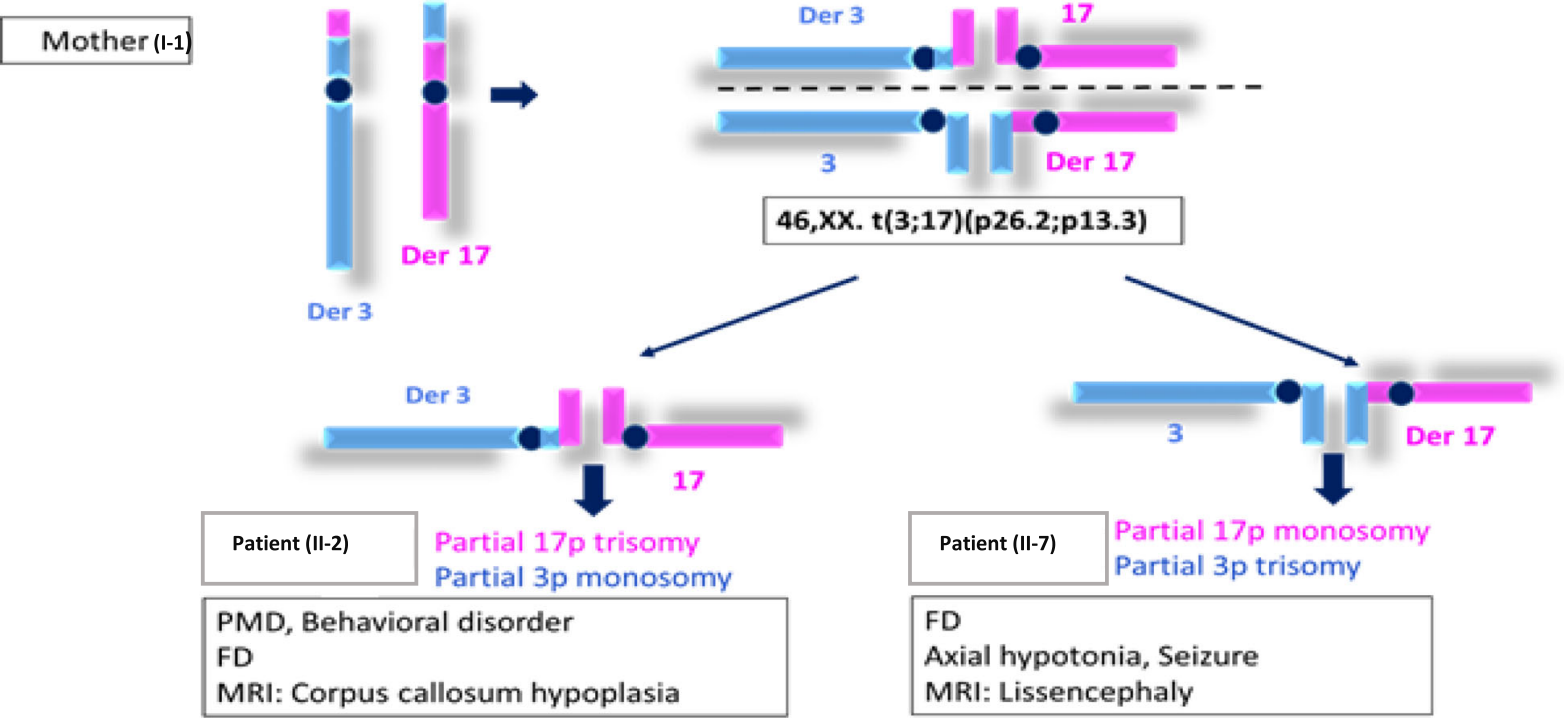
Ideograms of maternal chromosomes 17 and 3 and their derivatives der(17) and der(3)

FISH was first performed on the sister (II-7) using the subtelomeric probes (Vysis) of chromosome 17p and showed the absence of a subtelomeric signal on one of the chromosomes 17p (Fig. 3a). This was indicative of a family subtelomeric translocation (Fig. 4). Consequently, using the same probe of chromosome 17p, FISH analysis showed hybridization on the derivative chromosome 3 and on normal chromosome 17 (Fig. 3b), 46,XX.ish t(3;17)(p26.2;p13.3)(*LIS1*+,subtel3ptel+,subtel3qter+) in the mother. FISH was then performed in the proband (II-2) using 17p probe and showed three signals on the two normal chromosomes 17 and the derivative chromosome 3 (Fig. 3c). This confirmed the duplication of the terminal region of chromosome 17.

Ideograms of maternal chromosomes 17 and 3 illustrate the exchange of chromosome material of 17ptel and 3ptel regions due to the reciprocal translocation t(3;17). The patient (II-2) inherited the der(3) mat and the normal paternal chromosomes 17 and 3. The patient (II-7) inherited the der(17) mat and the normal paternal chromosomes 17 and 3.

**Fig. 5:**
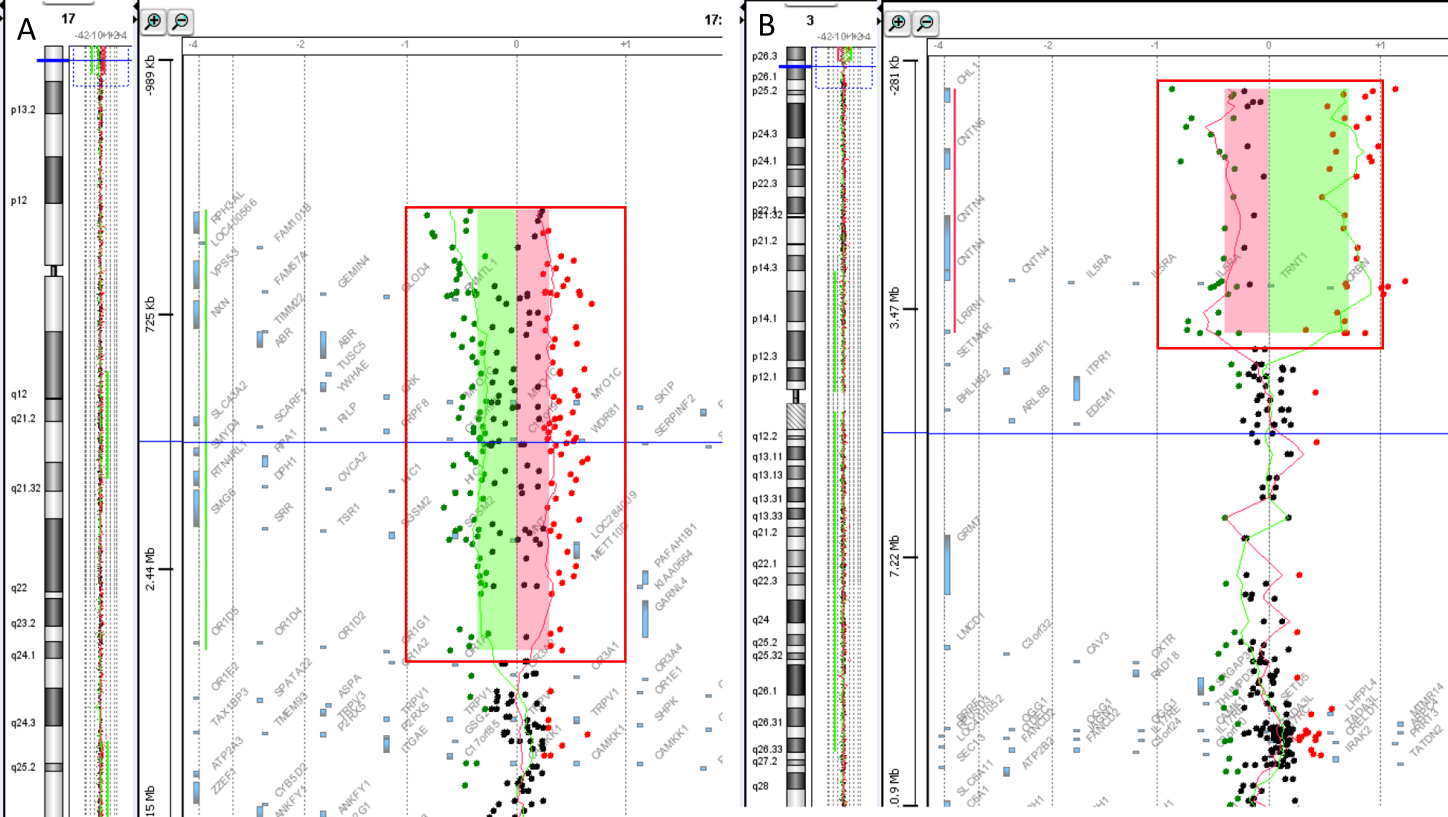
Results of 44 K Agilent oligo array-CGH analysis in patient II-2. **A.** chromosome 17, showing 17p13.3 duplication of at least 2,9 Mb in size. Chromosome 3, showing 3p26.2 deletion of at least 3,6 Mb in size

Aiming to delimit the involved segments, array-CGH analysis was performed on the proband and showed a large deletion of 3,6 Mb on the short arm of chromosome 3, involving 12 OMIM genes and a large duplication of 2,9 Mb on the short arm of chromosome 17, encompassing 61 OMIM genes: 46,XX.arr[GRCh18]3p26.2(224727_3864822)X1,17p13.3(48539_2976723)X3 mat (Fig. 5).

## Discussion

Adjacent-1 segregation of the translocation t(3;17) in the mother led to two different chromosome imbalances in the children. The first adjacent-1 type gave rise to a derivative 3 (der3) in patient II-2 that resulted in partial monosomy 3p and partial trisomy 17p*.* On the other hand, the second adjacent-1 type led to a derivative 17 (der17) in patient II-7, thus resulting in partial monosomy 17p and partial trisomy 3p. While deletions of 17p13.3 are associated with a well-known phenotype ranging from Miller Dieker syndrome [3] to partial agenesis of corpus callosum and milder phenotype [8], duplications of the same chromosomal region still need further clinical and molecular characterization. According to the involved genes, 17p13.3 duplications have been divided into either class I or class II leading to different clinical features [2].

**Fig. 6:**
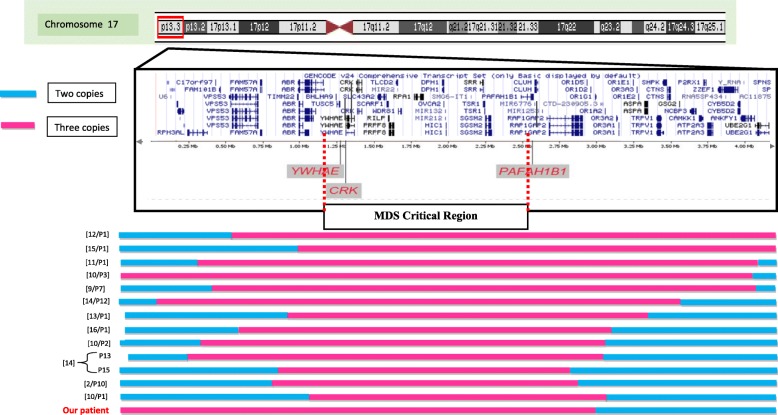
Schematic illustration of the molecular findings in individuals reported with duplication in the Miller Dieker Syndrome (MDS) Critical Region encompassing both *YWHAE* and *PAFAH1B1* genes

So far, to the best of our knowledge, only 13 patients having large 17p13.3 duplications, including the entire MDS comprising both *PAFAH1B1* and *YWHAE* genes have been reported [2, 9–15] (Fig. 6) with varying sizes and different breakpoints. It has also been reported that these duplications might be the result of parental translocations. They have never involved the 3p26 region.

The genomic distances (in base pairs from the 17p telomere) shown at the top of the figure were measured according to ensembl genome browser 59 (hg18). For each patient, a normal copy number is illustrated as a blue line and the duplicated segment as a pink line.

Here, our proband showed a loss of nearly 3,6 Mb on 3p26.2 and a gain of nearly 2,9 Mb on 17p13.3 and shared clinical and dysmorphic features including a high forehead and a triangular chin described in thirteen selected patients with duplication of the MDS region (Table 1). Our patient did not share some of these features whereas he presented arachnodactyly, which is rarely described in patients with partial trisomy of 17p13.3 [2, 9, 11, 16]. The most frequent phenotypic features associated with partial trisomy of 17p13.3 were correlated with the duplication of the *PAFAH1B1* and *YWAHE* genes that were located in the MDS region. It was hypothesized that the duplication of *YWHAE* might have an effect on neuronal network development and maturation, and was related to mild development delay and facial dysmorphisms while the duplication of *PAFAH1B1* that lead to its overexpression, was associated with moderate to severe development delay and structural brain abnormalities [2, 9]. Brain-imaging analysis was performed in seven of the eleven reported patients and only four showed structural brain abnormalities (Table 1), among which Corpus Callosum hypoplasia or agenesis represented the main brain abnormality [9, 10, 13, 14]. Likewise, our patient presented corpus callosum hypoplasia. Curiously, patients reported so far as having the smallest and the largest duplications of the MDS region present normal Magnetic Resonance Imaging (MRI) (P1/ [10]; P1/ [15]). This suggests that this heterogeneity depends on the size of the duplication and the involved genes as well as on the involvement of other gene interactions and modifier genes. Indeed, it has been proven that transgenic mice with increased lis1 expression in the developing brain revealed abnormalities in the neuroepithelium such as the thinning of the ventricular zone, and the ectopic positioning of mitotic cells [9]. Furthermore, lis1 overexpression affected both radial and tangential migration with a migration delay in radial migration at E13.5 and tangential migration at E12.5 rather than E14.5 [10]. However, subtelomeric neuronal migration defects are not expected to be detected by MRI scans [9]. Consequently, we can postulate that the overexpression of *LIS1* gene could account for the phenotype of our patient particularly corpus callosum hypoplasia.

**Table 1:** Comparison of the phenotypic features of the proband with patients showing duplication of Miller-Dieker region

Paper	[12]	[15]	[11]	[10]	[9]	[14]	[13]	[16]	[10]	[14]	[14]	[2]	[10]	Present Study
Patient reference	Patient 1	Patient 1	Patient 1	Patient 3	Patient 7	Patient 12	Patient 1	Patient 1	Patient 2	Patient 13	Patient 15	Patient 10	Patient 1	Patient 1
Size of duplication, Mb	10,7	5,77	4,2	4	3,6	3,4	3,22	3,1	3	2,78	2,16	2	1,8	2,9
Inheritance	Maternal balanced translocation	De novo	?	De novo	De novo	De novo	Paternal balanced translocation	Maternal balanced translocation	De novo	Paternal	De novo	De novo	De novo	Maternal balanced translocation
Age at diagnosis, years	prenatal	4	13	1	10	28	0.5	6	1	13mo	14	6.5	14	2
Gender	F	F	F	M	F	F	F	F	F	M	F	M	M	F
Birth height, cm	NA	55	Normal	50	53	NA	51	NA	NA	NA	NA	Normal	53	52
Birth weight, g	NA	2680	Normal	3380	3060	NA	3000	NA	4200	NA	NA	Normal	3350	3500
Current height	NA	+ 1SD	+ 1SD	+ 1SD	+ 1SD	NA	50–75th percentile	111 cm (10–25th percentile	Normal	NA	NA	Normal	+ 3.5 SD	+1,05DS
Current weight	NA	+1SD	+1SD	+1SD	+2SD	NA	25th percentile	17 kg (10th percentile)	-2SD	NA	NA	Normal	+1SD	+ 0,6DS
Cranio-facial dysmorphism Hypotonic face	NA	+	+	+	–	+	–	–	+	+	NA	NA	+	–
Broad midface	NA	NA	+	+	–	–	–	+	+	–	–	NA	–	–
High forehead	+	+	–	+	–	NA	–	+	+	+	NA	NA	+	+
Upward palpebral fissures	NA	+	–	–	+	NA	+	+	–	–	NA	–	–	+
Hypertelorism	NA	+	+	+	–	–	+	+	+	–	–	–	+	+
Epicanthus	NA	NA	NA	+	NA	NA	–	–	–	–	NA	NA	–	+
Strabismus	NA	NA	–	–	+	NA	+	–	–	–	NA	–	–	–
Broad nasal bridge	NA	+	+	+	–	NA	+	+	+	+	NA	–	+	+
Small mouth	NA	+	+	+	Normal	+	+	+	+	+	+	Prominent cupid bow	Normal	+
Low-set-ears	+	NA	–	–	–	NA	–	+	+	+	NA	NA	+	–
Triangular chin	NA	NA	+	+	NA	+	+	–	+	+	+	+	–	+
Neck appearance	NA	NA	Normal	Short	Normal	NA	Short	Normal	Short	NA	NA	Normal	Normal	Short
Limb abnormalities	NA	NA	+	–	–	–	Long fingers	Long fingers	+	–	–	–	–	Long fingers
Hip luxation	NA	NA	–	+	–	NA	–	–	–	NA	NA	–	–	–
Equinovalgus	NA	NA	–	Right	–	NA	+	–	–	NA	NA	–	–	–
Neurological features Hypotonia	NA	+	+	+	–	NA	–	–	+	–	+	+	+	–
Delayed mental development	NA	+	+	+	+	LD	+	–	+	Mild LD	Mild LD	–	+	–
Delayed motor development	NA	+	+	+	+	+	+	+	+	NA	+	–	+	+
Abnormal behavior	NA	NA	+	+	+	+	+	+	+	–	–	Autism	+	+
Brain imaging results	NA	Normal	Normal	Dilated lateral ventricles/ Corpus Callosum Agenesis	Reduced brain size, Corpus Callosum Hypoplasia, Cerebellar Agenesis	NA	Cortical Atrophy and Hypoplasia of Corpus Callosum	NA	NA	Thin Corpus Callosum, Cerebellar vermis hypoplasia	NA	NA	Normal	Corpus Callosum Hypoplasia

+: present/−:absent/NA:not available

Numerous features in this case might be attributed to genes that are lost in chromosome 3p in addition to 17p13.3 duplication as a result of adjacent-1 malsegregation of the maternal balanced translocation. In fact, it has been shown that terminal 3p deletions are responsible for a rare contiguous gene disorder (OMIM# 613792) [17]. Interestingly, we reviewed six previously reported cases having 3p deletion, compared them to the present case report, and noted that the most frequent features are microcephaly, corpus callosum hypoplasia and facial dysmorphia [18, 19] (Table 2). Conversely, some studies reported cases with 3p deletion and normal phenotypes [17, 22, 23]. In other studies, the authors have even hypothesized that distal 3p deletion is probably associated with normal intelligence and normal physical features [18, 24] and that the severity of the phenotype depends on the size of the deletion as well as on the gene content and the disrupted genes involved in the breakpoints, essentially *CNTN4, CNTN6* and *CRBN* [25, 26]. The *CNTN6* gene plays a crucial role in the development, maintenance, and plasticity of functional neuronal networks in the central nervous system. It has been shown that *Cntn6* deficiency in mice causes profound motor coordination abnormalities and learning difficulties [25]. Owing to its function, we suggest that *CNTN6* gene could be responsible for the observed psychomotor development retardation in the current case. On the other hand, *CNTN4* is known to be involved in axon growth, guidance, and fasciculation [25] and it probably contributes to the behavioral abnormalities in our patient showing aggressiveness, anger and agitation. In fact, cntn4 knockout mice showed morphological, neurological and behavioral abnormalities [25]. The deletion also included *CRBN* gene that plays a crucial role in brain development [26]. In fact, CRBN protein is part of the DCX protein ligase complex involved in the regulation of the surface expression of certain types of ion channels in neuronal memory synapses. Furthermore, 3p26 deletion disrupted a more distal gene: *CHL1* that plays a crucial role in the development of the cortex by regulating neuronal differentiation and axon guidance [27]. Previous studies suggested *CHL1* as a dosage-sensitive gene with a major role in intellectual disabilities [28]. Interestingly, Frints hypothesized that a reduction equal to 50% of chl1 in the developing brain marked cognitive deficit [29].

**Table 2:** Comparison of the phenotypic features of the proband with patients showing 3p26 deletion

Paper	[4]	[18]	[20]	[21]	[19]	[17]	Present Study
Patient reference	Patient 1	Patient 1	Patient 2	Family F	Patient 1	Patient 1	Patient 1
Size of deletion, Mb	4,5	1,5	1,05	2,95	7,4	2,9	2,9
Inheritance	De novo	Paternal	Maternal balanced translocation	?	?	Maternal	Maternal balanced translocation
Age at diagnosis, years	16	9	24	14	prenatal	1 and 2 months	2
Gender	M	M	M	M	F	M	F
Birth height, cm	71	123	58	140	NA	48	52
Birth weight, g	2695	2600	5350	3400	295	3000	3500
Current height	NA	NA	-2SD	NA	NA		+1,05DS
Current weight	NA	NA	-2SD	NA	NA		+ 0,6DS
Cranio-facial dysmorphism	+	NA	+	+	+	+	+
Upward palpebral fissures	NA	NA	NA	+	NA	NA	+
Hypertelorism	+	NA	NA	NA	+	NA	+
Blepharophimosis	+	NA	NA	NA	NA	NA	NA
Eyelid	+	+	NA	NA	NA	NA	NA
Broad nasal bridge	+	NA	+	+	+	+	+
Micrognathia	+	NA	NA	NA	+	NA	
Low-set-ears	+	NA	+	+	+	NA	–
Short philtrum	–	NA	+	+	+	NA	+
Limb abnormalities	–	–	–	bilateral clinodactyly of the fifth finger	NA	NA	+
Ptosis	+	+	NA	NA	+	NA	–
Microcephaly	+	+	+	+	brachycephaly	+	+
Neurological features Hypotonia	+	+	+		NA	NA	–
Delayed mental development	+	+	+	+	NA	–	–
Delayed motor development	NA	NA	+	+	NA	NA	–
Abnormal behavior	NA	NA	NA	Hysterical and aggressive	NA	NA	+
Brain imaging results	A	Centrotemporal spikes in the left hemisphere	Corpus callosum hypoplasia	NA	NA	Corpus callosum dysgenesis	Corpus Callosum Hypoplasia

+: present/−:absent/NA:not available

Interestingly, both 3p deletion and17p duplication could share the same network in neuronal migration since both anomalies lead to corpus callosum hypoplasia and pachygyria. So far, both *PAFAH1B1*genes duplicated in 17p and *CNTN6* as well as *CRBN* genes deleted in 3p affected the process of cortical development by destabilization of microtubules and alteration of axon growth and axon guidance [25, 30, 31].

Neuronal migration is a complex process that involves several actors and factors in order to elaborate an appropriate cell migration from the ventricular zone into the cortical plate during normal brain development [32]. Mutations and chromosomal aberrations can alter chromosome 3D organization. This alteration could play a more important role than we believe it does in the chromosomal interactions and transcriptional regulation of genes. In fact, it has been shown that chromatin 3D modification could disturb the topologically associating domains (TADs) and consequently the regulation of gene expression [33]. Such alteration could explain the phenotypic variability in human disease ranging from a milder phenotype to a microdeletion/microduplication syndrome. Furthermore, this variability can be explained by the consanguinity in this family, which reduces the suitability of individuals by increasing the degree of homozygosity and promoting the development of deleterious recessive genes [34]. Finally, patients carrying CNVs known to have broad variable clinical expressivity and possibly incomplete penetrance, may benefit from whole exome sequencing analysis in the near future.

## Conclusions

The variability of genes, which are mapped in the involved regions (3p and 17p), and the description of the clinical characteristics of our patient contribute to the confirmation and further delineation of the associated characteristics to the partial trisomy of 17p13.3 encompassing the entire MDS critical region as well as the partial monosomy of chromosome 3p26.2. Various genes and structural chromosomal anomalies have been discovered as being involved in this process. However, the exact molecular basis of brain malformations still needs further studies.

## Data Availability

The datasets generated and/or analyzed during the current study are available in the [ArrayExpress] repository. [https://www.ebi.ac.uk/arrayexpress/experiments/E-MTAB-8748].

## Abbreviations

Array CGH: Array comparative genomic hybridization
*CHL1*: close homolog of L1
*CNTN4*: contactin 4
*CNTN6*: contactin 6
*CRBN*: cereblon
ISCN: International System for Human Cytogenetic Nomenclature
OMIM: Online Mendelian Inheritance in Man
*PAFAH1B1*: platelet activating factor acetylhydrolase 1b regulatory subunit 1
SD: standard deviation
*YWHAE*: tyrosine 3-monooxygenase/tryptophan 5-monooxygenase activation protein epsilon
